# Effect of axitinib regulating the pathological blood–brain barrier functional recovery for glioblastoma therapeutics

**DOI:** 10.1111/cns.13788

**Published:** 2021-12-29

**Authors:** Fengtian Zhang, Lijuan Wen, Kai Wang, Zhihua Huang, Xiangyu Jin, Ruiwen Xiong, Shiying He, Fuqiang Hu

**Affiliations:** ^1^ Medical College of Soochow University 199 Renai Road, Suzhou Industrial Park Suzhou China; ^2^ Department of Orthopedics First Affiliated Hospital of Gannan Medical University Ganzhou China; ^3^ College of Pharmaceutical Sciences Zhejiang University Hangzhou China; ^4^ National Engineering Research Center for Modernization of Tranditional Chinese Medicine‐Hakka Medical Resources Branch College of Pharmacy Gannan Medical University Ganzhou China; ^5^ Department of Physiology School of Basic Medical Sciences Institute for Medical Sciences of Pain Gannan Medical University Ganzhou China; ^6^ Key Laboratory of Prevention and Treatment of Cardiovascular and Cerebrovascular Diseases of Ministry of Education Gannan Medical University Ganzhou China

**Keywords:** blood–brain barrier, glioblastoma, pathological disruption, restoration, therapeutics

## Abstract

**Aims:**

Dysfunction of the blood–brain barrier (BBB) is a prominent pathological feature of glioblastoma (GBM). Vascular endothelial growth factor (VEGF) is confirmed to be abnormally elevated in the pathogenesis of GBM, causing BBB pathological disruption, which further allows the leakage of neurotoxic blood‐derived molecules into the central nervous system (CNS), interfering brain homeostasis and leading to poor patient outcome. Since BBB is an integral and pivotal part of the brain microenvironment, which strongly supports the occurrence and the pathological progression of GBM, here we have selected the VEGFR antagonist axitinib as a BBB functional regulator and hypothesized to regulate pathological BBB restoration for GBM effective treatment.

**Methods:**

The pathological BBB cell model was constructed to investigate the timeliness and dose effect of axitinib regulating pathological BBB restoration. In order to investigate the efficacy and safety of axitinib regulating pathological BBB restoration for anti‐GBM treatment, the orthotropic GBM‐bearing mice model was established for in vivo study, and bioluminescent imaging was used to real‐time and noninvasively monitor tumor growth response in vivo, and survival time was also recorded.

**Results:**

Axitinib under non‐cytotoxic dosage regulated pathological BBB restoration in a time‐dependent mode, and multiple intervention of axitinib could realize a visible restoration of pathological BBB in vitro. Moreover, axitinib treatment restored pathological BBB in orthotropic GBM‐bearing mice. We further confirmed that functional restoration of pathological BBB with axitinib had certain curative effect in prolonging median survival of orthotropic GBM‐bearing mice at non‐cytotoxic dosages in vivo.

**Conclusion:**

The mechanism of axitinib involved in BBB functional regulation in the treatment of GBM is first illuminated in this report; moreover, this is the first report first referring to regulating pathological BBB functional recovery for GBM effective therapeutics. Overall, the view of regulating pathological BBB functional recovery may offer a novel sight for other CNS diseases relating to BBB permeability effective therapeutics.

## Graphical abstract

The occurrence of glioblastoma (GBM) induces blood brain barrier (BBB) pathological damage, and the restoration of pathological BBB induced by axitinib could produce considerable anti‐GBM effect.

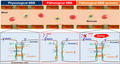

## 1 INTRODUCTION

Glioblastoma (GBM) is a kind of malignant brain tumor with high mortality and no curative treatments.^1^ Despite advances in molecular understanding, diagnosis and clinical standard therapies (surgery, chemotherapy, and radiotherapy), the overall median survival of patients with GBM has only been extended to 14 ~ 16 months.^2^ In the last decades, GBM has been merely one of a few from the great number of central nervous system (CNS) pathologies benefiting from advancements in the modern neurology research. Even though the etiology and pathogenesis of the disorder is known, there is still no fully functional treatment to slow its progression. The presence of the blood–brain barrier (BBB) seriously limits the trafficking of most molecules, including 98% of small‐molecule drugs and most bio‐macromolecular drugs, to and from the brain and limits the therapeutic effect of brain diseases.^3^, ^4^

In recent years, the receptor‐mediated active‐targeted drug delivery system was considered one of the most mature and valid strategies for GBM therapeutics due to its characteristics of high specificity, selectivity, and affinity in the field of pharmaceutics.^5^, ^6^ However, so far, brain targeted technology research has mostly focused on the interaction between ligands and their receptors and target cells and target tissues, ignoring the correlation between the changes of BBB function and disease treatment.^7^, ^8^ Our previous studies have found that the regulation of BBB function could promote more chemotherapeutics distributed or targeted at GBM, finally realizing effective and safe anti‐GBM effect of chemotherapy.^9^ Therefore, the innovation of pharmaceutics research methods is especially needed for some clinically intractable or even drug‐free CNS diseases. And, the treatment strategy of BBB functional regulation may be more therapeutically valuable and can be put into clinical application more quickly. At present, regulation of BBB function has been one of the latest development directions in molecular pharmaceutics.

The focal microenvironment strongly supports the occurrence and the pathological progression of diseases.^10^ Recently, the concept of microenvironment regulation may be a new therapeutic approach that is worth exploring, and there is tremendous worldwide interest in novel therapeutic strategies aiming at such special conditions. For example, the destruction of various stromal cells and extracellular matrix components in the tumor stromal microenvironment, to further rebuild the tumor stromal microenvironment and enhance the accessibility of drugs to tumor cells, would significantly inhibit the growth of peripheral cancers.^11^ Due to the special growth location of GBM, the BBB is involved in the formation of the special brain microenvironment of the disease.

The BBB plays an important role in the occurrence and treatment of GBM. Under physiological conditions, the BBB is an important biological barrier to protect and maintain the physiological function of the normal brain and the environment balance of the CNS.^12^ Under pathological condition, the occurrence of GBM is usually accompanied by BBB dysfunction.^13^ Moreover, the loss of BBB integrity causes the leakage of harmful substances in the blood into the CNS, forming cell infiltration, abnormal molecular transportation and clearance, and accelerating the pathological deterioration of GBM, leading to poor prognosis.^13^, ^14^ Previous studies have indicated that a decreased permeability of the pathological BBB in the period of therapeutics treatment could predict a good prognosis.^9^, ^15^ Hence, we hypothesized that pathological BBB restoration might provide new opportunities for GBM safe and effective therapeutics.

Tight junctions (TJs) between cerebral microvascular endothelial cells is an important morphological basis of the BBB and forms low permeability of the barrier.^4^ Furthermore, claudin‐5 and occludin are key components of TJs and play an important role in maintaining the integrity and function of the BBB.^8^, ^9^ Our previous studies have indicated that the VEGF/VEGFR signaling pathway was involved in BBB permeability regulation in the occurrence of GBM, and the activation of the VEGF downstream signaling pathway PI3K‐AKT could obviously open the BBB for enhanced intracranial drug delivery with a visible improved anti‐GBM effect.^9^ Herein, we further proposed to inhibit the interaction between VEGF and its receptor VEGFR to finally realize a functional restoration of the pathological BBB for anti‐GBM therapeutics.

Axitinib, a potent small‐molecule tyrosine kinase inhibitor (TKI) that could selective inhibit the VEGF receptors 1, 2, and 3, has been approved for use in advanced renal cell carcinoma.^16^ In recent years, studies have found that axitinib exhibits therapeutic effect on non–small cell lung cancer and leukemia.^17^, ^18^ In addition, it has also been found that axitinib can resist high‐fat diet–induced obesity and insulin insensitivity by promoting the browning of white fat cells, and has the effect on treating fatty liver.^19^ However, the effect of axitinib regulating BBB permeability for GBM therapeutics has not yet been explored. Here, axitinib was used as a BBB functional regulator, and studies were performed to address the effect of axitinib restoring pathological BBB for the treatment of GBM within non‐cytotoxic dosages.

## 2 MATERIALS AND METHODS

### 2.1 Materials

Axitinib was purchased from Selleck. Methylthiazoleterazolium (MTT) was provided by Sigma Chemical Co. FITC‐dextran (10 kDa) was purchased from Sigma‐Aldrich Co. D‐luciferin was purchased from Shanghai Sciencelight Biology Science & Technology Co., Ltd. All of the other chemicals used in this article were of analytical chromatographic or analytical grade.

### 2.2 Cell models and animal models

Both bEnd.3 cells and U87 MG cells without mycoplasma were purchased from the Cell Bank of ATCC. bEnd.3 cells and human GBM cell line U87 MG cells were maintained in Dulbecco's minimum essential medium (DMEM) and α‐MEM (Gibco), respectively, both supplemented with 10% fetal bovine serum (FBS) (Gibco) and 100 IU/ml penicillin‐streptomycin at 37℃ in a 5% CO_2_. The cells were sub‐cultured regularly using trypsin/ethylene diamine tetraacetic acid (EDTA).

The physiological BBB model and pathological BBB model in vitro^9^ and the orthotopic GBM‐bearing mice models were established according to previous methods.^8^, ^9^ The detailed methodology was provided in Supplementary Materials.

### 2.3 Cytotoxicity assay

The MTT assay was used to verify the cytotoxicity effect of axitinib on both bEnd.3 cells and U87 MG cells. And, the detailed methodology is provided in Supplementary Materials.

### 2.4 Dose‐curve and time‐curve of axitinib restoring the pathological BBB in vitro

The cells in the Transwell chambers were treated with axitinib within a series of concentrations (0–10 μg/ml) to stimulate the pathological BBB in vitro. And, the transepithelial electrical resistances (TEER) values were used to evaluate BBB integrity. For time‐curve investigation, the pathological BBB was treated with optimal axitinib dosage every 24 h for 5 consecutive days, and the TEER was real‐time monitored (0 h, 24 h, 48 h, 72 h, 96 h, 120 h, and 144 h) for BBB permeability evaluation.

### 2.5 Pathological BBB functional recovery with the treatment of axitinib in vitro

We considered claudin‐5 and occludin as markers of intact TJs.^13^ The pathological BBB was treated with appropriate concentrations of axitinib for 24 h. For comparison, the bEnd.3‐U87 MG co‐cultured model without any treatment was considered as negative controls of brain tumor condition, and the physiological BBB model was considered positive control. For qualitative research, the expression of both claudin‐5 and occludin proteins on bEnd.3 cells was investigated by immunofluorescent staining, and the detailed method was provided in Supplementary Materials. And, the measurement of TEER and transportation of FITC‐dextran (10 kDa) crossing the barrier were further used to quantitatively determine the permeability of the BBB in vitro.

### 2.6 Axitinib promotes pathological BBB functional recovery in vivo

We then used Evans Blue (EB) to study pathological BBB recovery caused by axitinib in orthotropic GBM models.^9^, ^20^ Axitinib (10 mg/kg) was I.V. administered to orthotropic GBM‐bearing mice; then, EB was intravenously injected, and normal animals served as the control. Four hours later, the animals were perfused with 4% paraformaldehyde to flush the intravascular dye out of the vasculature. The imaging of excised brain tissues was observed under an in vivo imaging system (CRI).

### 2.7 Regulation of pathological BBB functional recovery for GBM therapy in vivo

For pathological BBB restoration in GBM therapy, GBM‐bearing model mice were randomly divided into two groups: saline group and axitinib treatment group (*n* = 10). Axitinib was I.V. administered with a dose of 10 mg/kg for 7 consecutive days. Bioluminescence imaging (IVIS spectrum) was used to noninvasively monitor tumor growth, and the images were obtained on day 4, 7, 10, 13, and 17 post inoculation. Furthermore, the quantitative total bioluminescence was determined by drawing regions of interest (ROIs) around tumor areas enclosing emitted signals (*n* = 5). After the treatment, three mice of both the saline group and axitinib treatment group were sacrificed, and brains and other major organs were harvested for routine histopathological and immunohistochemical (IHC) analysis. All mice were euthanized when they became moribund, and survival time was recorded (*n* = 6) by using the log‐rank test in the Kaplan–Meier analysis method (SigmaPlot). Body weight of saline‐treated mice and axitinib treated mice was also recorded.

In order to investigate VEGF expression in vivo, tumor tissues were processed for total mRNA or protein expression followed by qRT‐PCR and IHC, respectively. Moreover, TJ proteins of claudin‐5 and occludin expression both on the gene level and protein level were determined by qRT‐PCR and immunofluorescent staining, respectively. IHC of CD31 and MMP‐9 in brain tumor tissues was also performed.

### 2.8 Evans Blue extravasation

Blood–brain barrier integrity was also studied in the experimental mice. We intravenously injected EB (10 mg/kg) dye into normal or GBM‐implanted mice. The tracer was allowed to circulate for 2 h before the mice were deeply anesthetized and perfused with PBS followed by 4% PFA. The imaging of excised brain tissues was also observed under the in vivo imaging system. Then, the EB in brain tissues was extracted with dimethyl formamide and then quantificationally measured by using an Ultraviolet Spectrophotometer. Brain tissues were dried under 60℃ for 72 h.

### 2.9 Statistical analysis

All results in this article were repeated in triplicate unless otherwise stated; the declared group size is number of independent values, and quantitative data were expressed as mean ± SD analyzed by GraphPad Prism 7.0. For two group comparisons, statistical significance was determined by Student's *t*‐test. Besides, the log‐rank test in the Kaplan–Meier analysis method was used to record survival time of experimental animals. The statistical significance was defined as a *p*‐value less than 0.05, and all tests were two‐tailed. ***p < 0.001, **p < 0.01, and *p < 0.05 were performed in one‐way ANOVA (*t*‐test).

## 3 RESULTS

### 3.1 Dose‐curve of axitinib restores the pathological BBB

We first explore the cytotoxicity of axitinib against model cells (bEnd.3 cells and U87 MG cells). As shown in Figure S1, survival rates of both bEnd.3 cells and U87 MG cells were above 80% even when the concentration of axitinib reached 10 μg/ml. The result indicated that axitinib exhibited negligible cytotoxicity to model cells at experimental doses. In order to avoid the cytotoxicity interference of axitinib, we used axitinib at concentration ≤10 μg/ml for further BBB functional regulation studies.

**FIGURE 1:**
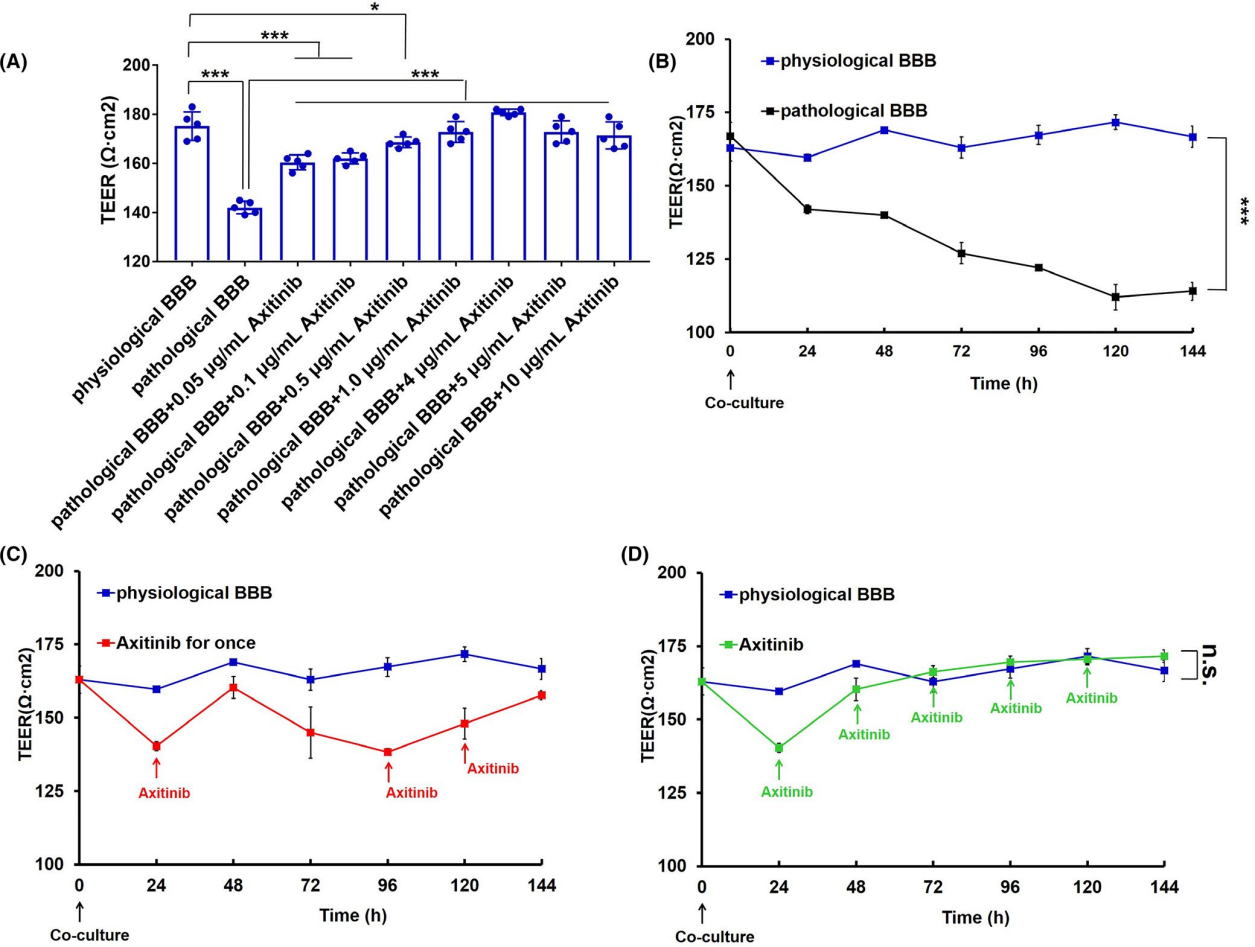
Dose‐curve and time‐curve of axitinib restoring the pathological BBB in vitro. (A) TEER measurement of the pathological BBB with a series of concentrations of axitinib (0–10 μg axitinib‐equiv./ml) for 24 h. TEER measurement of (B) the pathological BBB, (C) with a single treatment of axitinib for the first time, 72 h later for the second time and 96 h later for the third time (red solid arrows indicated the stimulation of axitinib), and (D) with a consecutive treatment of axitinib for every 24 h (green solid arrows indicated the stimulation of axitinib). Error bars represent SD of the mean for *n* = 5

We then investigated the effect of axitinib on pathological BBB functional regulation under a series of concentrations. TEER is usually used to verify the integrity of the cell monolayer in vitro, and here TEER measurement was conducted to evaluate BBB integrity and permeability. As shown in Figure 1A, the TEER of the pathological BBB seriously decreased compared to that of the physiological BBB, while the treatment of axitinib to the pathological BBB with a concentration of 0.05–10 μg/ml could effectively increase TEER values, and the optimal dosage of axitinib recovering the pathological BBB exhibited 4 μg/ml, which was then administered in the following in vitro experiments.

### 3.2 Time‐curve of axitinib restore the pathological BBB

Furthermore, the optimal intervention time of axitinib to the pathological BBB was also assessed through the measurement of TEER. As seem in Figure 1B–D, the co‐culture of bEnd.3 cells and U87 MG cells finally resulted in the formation of the pathological BBB with a serious reduction of TEER. For the first treatment with axitinib (Figure 1C, red solid arrow), the TEER rose and then began to decline 24 h later (Figure 1C, red line). Interestingly, 24 h after the first incubation of axitinib, the recovered BBB gradually returned to the pathological state, and a second axitinib administration could promote the restoration of the barrier again within 24 h. Moreover, if axitinib was administered every 24 h (Figure 1D, green solid arrow), the TEER continued to rise (Figure 1D, green line) and finally exhibited no difference with that of the physiological BBB (Figure 1B–D, blue line). In contrast, with the co‐culture time of bEnd.3 cells and U87 MG cells prolonged, the TEER of the pathological BBB model without any treatment (negative control group) continued to decrease (Figure 1B, black line). The phenomena indicated that the continued stimulation with axitinib could finally restore the pathological BBB, which provided sufficient theoretical guidance for further therapy of axitinib in vivo.

### 3.3 Axitinib restores the pathological BBB in vitro

**FIGURE 2:**
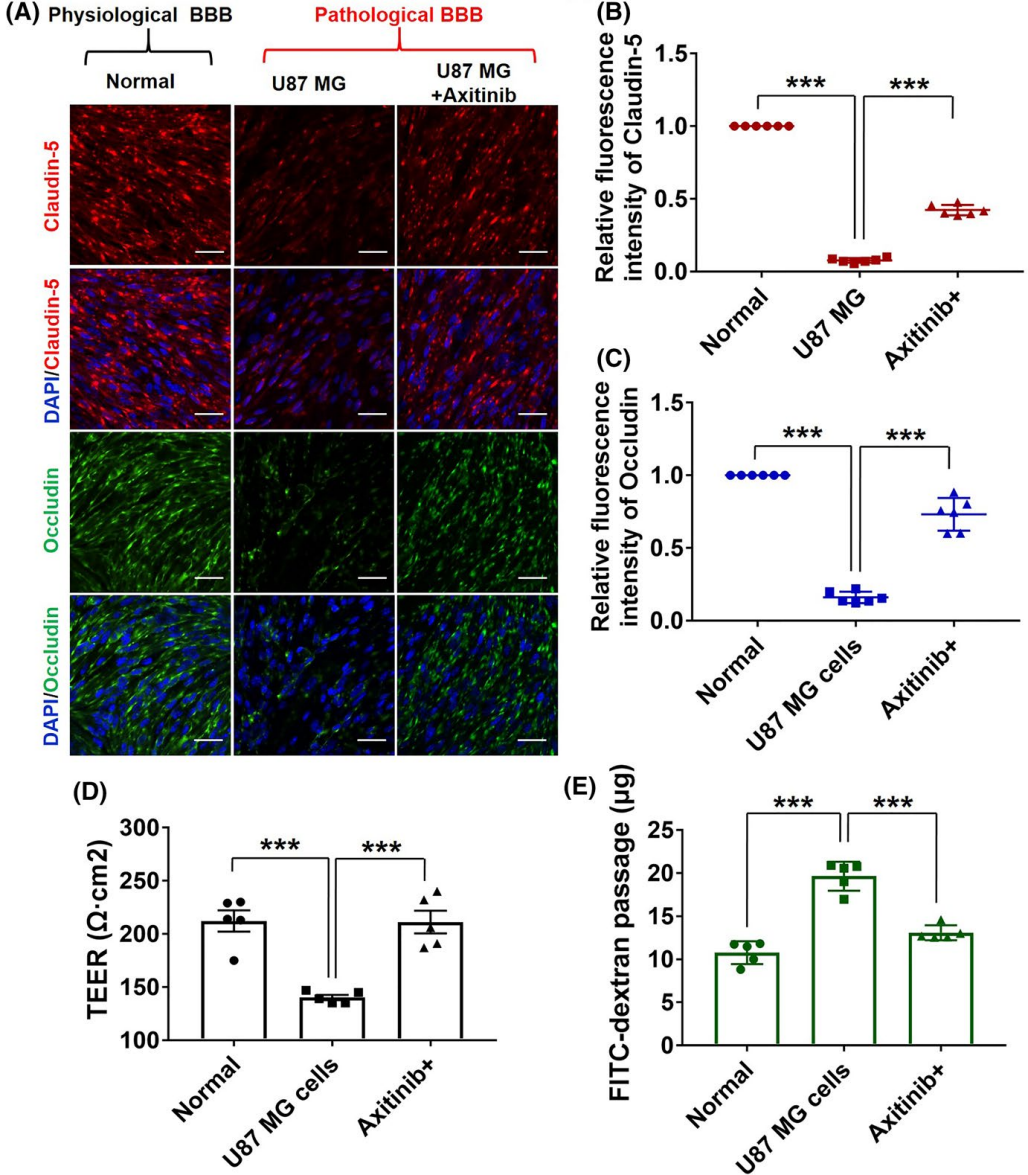
Axitinib restores the pathological BBB in vitro. (A) Claudin‐5 (red) and occludin (green) expression in the pathological BBB treated with or without axitinib (4 μg/ml) treatment (Scale bar = 30 μm). The fluorescence intensity of (B) claudin‐5 and (C) occludin signals measured by ImageJ. (D) TEER measurement of GBM cells induced the pathological BBB. (E) FITC‐dextran transportation across the pathological BBB with axitinib treatment

Glioblastoma growth causes BBB severe breakdown, accompanied with serious TJ structure breakdown and barrier pathological leakage.^9^, ^21^ Since claudin‐5 and occludin play a key role in the formation of TJs, here we consider them as representative markers of TJ pathological disruption and BBB dysfunction. As shown in Figure 2A, axitinib treatment significantly up‐regulated the expression of claudin‐5 (red) and occludin (green), and the fluorescence semi‐quantitative analysis of claudin‐5 (Figure 2B) and occludin (Figure 2C) further demonstrated that axitinib exhibited considerable ability to ameliorate the breakdown of the pathological BBB. In addition, pathological BBB restoration finally produced an obvious increased TEER (Figure 2D) from 140.2 ± 5.6 Ω cm^2^ to 211.2 ± 5.6 Ω cm^2^ and an evident reduced FITC‐dextran transporting across the BBB (19.8 ± 1.9 to 13.1 ± 0.9 μg/ml) (Figure 2E) in vitro. These results indicated that axitinib could promote the pathological BBB restoration in vitro.

### 3.4 Axitinib regulates pathological BBB functional recovery of GBM in vivo

**FIGURE 3:**
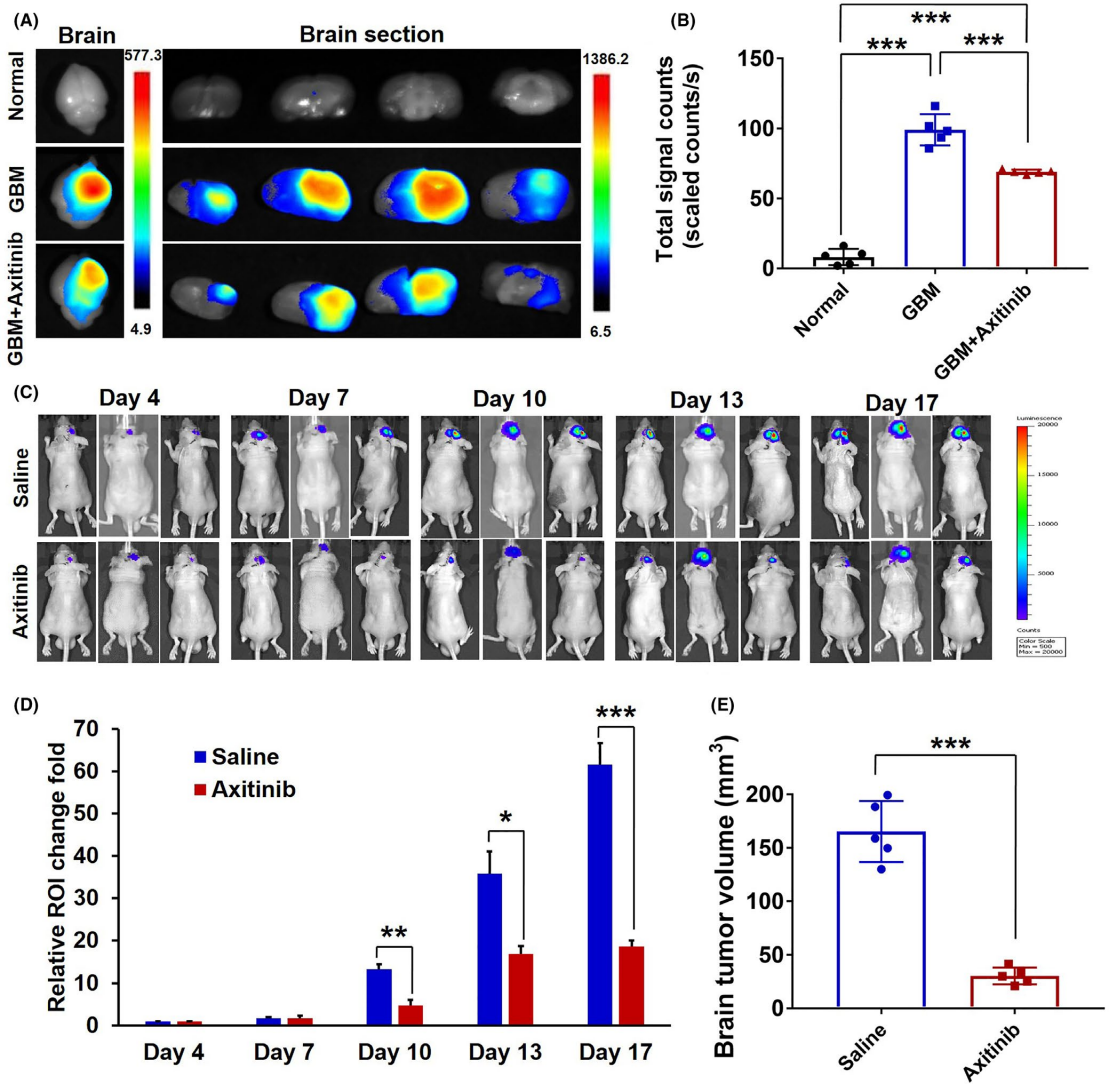
Restoration of the pathological BBB for GBM therapy in vivo. (A) Axitinib treatment induced the decrease of BBB permeability, EB extravasation in vivo was imaged, and (B) total signal count analysis of brain tissues. (C) Bioluminescence imaging of U87‐luci–bearing nude mice on days 4, 7, 10, 13, and 17 post U87‐luciferase tumor inoculation and (D) quantification of average tumor bioluminescence values change fold in different treatment formulations on days 4, 7, 10, 13, and 17 throughout the course of treatment (*n* = 5). (E) The final tumor volumes of experimental groups

The BBB is a highly specialized brain endothelial structure of the fully differentiated neurovascular system, restricting the free leakage of most large molecules and more than 98% of small‐molecule pharmaceuticals into the brain.^22^ The growth of GBM causes BBB pathological disruption and damages the homeostatic microenvironment of the brain, followed by blood component extravasation into the brain. Besides, BBB disruption can promote the proliferation of GBM to a certain extent.^21^, ^23^ The Evans blue (EB) dye is usually used as a classic indicator of BBB leakage in vivo due to its ability to bind the albumin of the plasma.^9^ Thus, we evaluated BBB permeability of the GBM‐bearing mice model via EB dye imaging in vivo. The ex vivo imaging of the brain tissues of mice in experimental groups is shown in Figure 3A. GBM growth significantly increases BBB permeability with an obvious EB extravasation into the brain, while axitinib intervention produced a significant decrease of EB leakage into the brain, accompanied with a decreased fluorescence intensity measurement in the brain (Figure 3B). The phenomenon further indicated that axitinib could promote pathological BBB functional recovery of GBM in vivo.

### 3.5 Restoration of pathological BBB for GBM therapy in vivo

**FIGURE 4:**
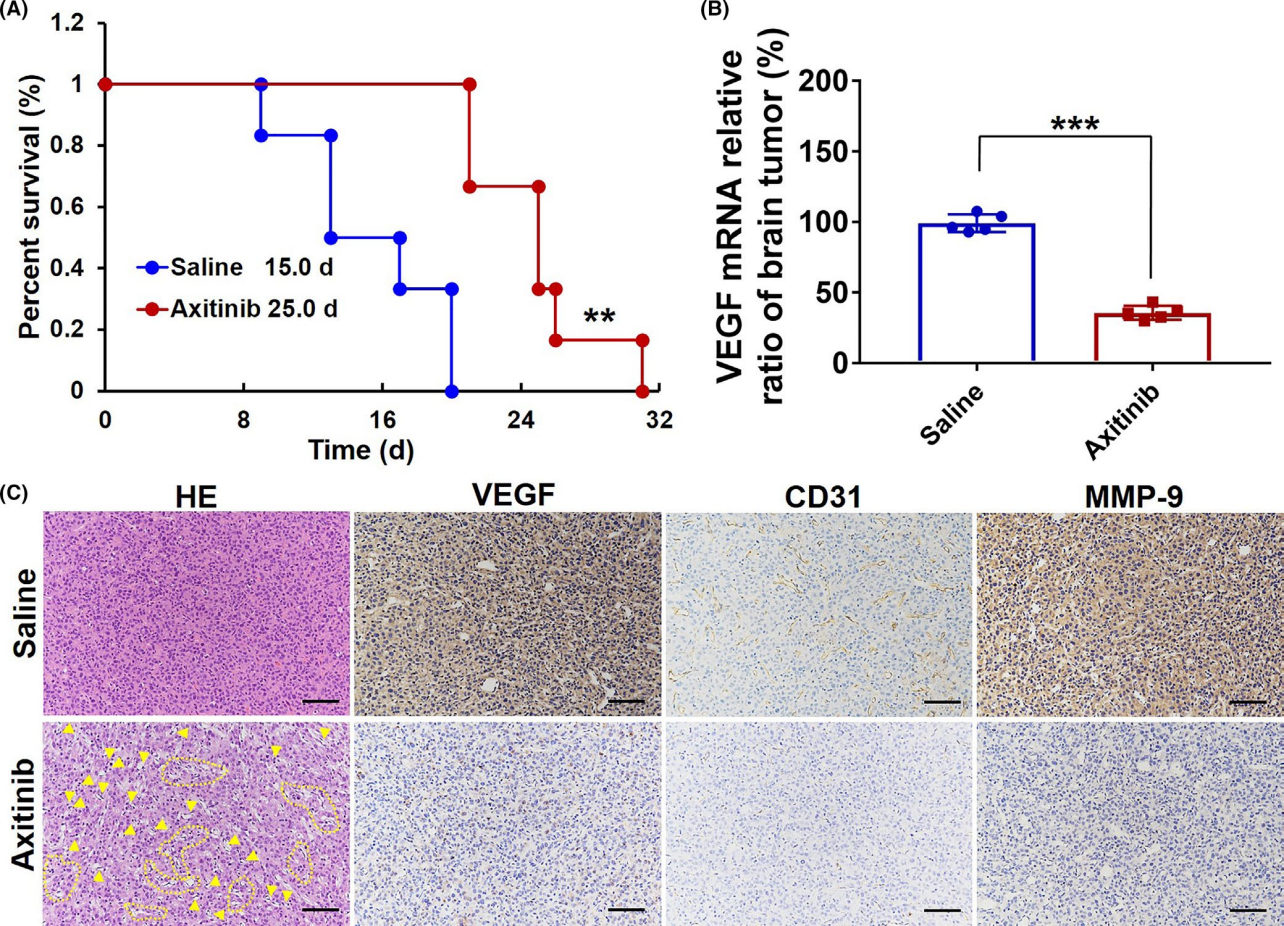
Tumor suppression in vivo. (A) Survival time of GBM‐bearing mice and log‐rank test in the Kaplan–Meier analysis method was used to record survival time of GBM‐bearing mice (*n* = 6). (B) VEGF mRNA expression of tumor tissues. Data were expressed as the fold change in VEGF expression normalized with the housekeeping gene, and GAPDH was amplified as an internal control. (C) H&E staining and IHC analysis of tumor sections stained with VEGF, CD31, and MMP‐9 (brown). Magnification: 200× (Scale bar = 100 μm)

To validate our hypothesis that the restoration of pathological BBB may provide a new sight for anti‐GBM therapeutics, we further evaluated the tumor suppression effect of axitinib in vivo. In this study, axitinib was I.V. administered with a dose of 10 mg/kg for seven consecutive days. Due to the specific growth site of the GBM, we here used bioluminescent imaging to real‐time and noninvasively monitor tumor growth response. The bioluminescent imaging of experimental groups is presented in Figure 3C, and relative fold changes in tumor bioluminescence over the course of drug treatment are presented in Table S1. Compared with the saline treatment group, the relative GBM growth rate of the axitinib treatment group decreased from 61.62 ± 4.94 to 18.66 ± 1.38 (Figure 3D and Table S1); moreover, the treatment of axitinib could achieve a visible tumor suppression effect (81.5%) (Figure 3E), finally translating into prolonged survival of GBM‐bearing mice. The survival curve span of the axitinib treatment group was extended from 20.0 days to 31.0 days, and the median survival of animals was prolonged to 167% (Figure 4A, red solid line). While for comparison, the median survival time of axitinib in the preclinical GBM model was just prolonged to 115%, even within an administrated dose of 25 mg/kg daily for 4 weeks.^24^

Moreover, no markedly body weight changes were found in the axitinib treatment group (Figure S2). In comparison, the body weight of the saline group decreased from 20.6 ± 0.1 g to 15.3 ± 0.2 g, with a serious weight loss of 25.7% on average during the treatment. Furthermore, axitinib treatment not only produced obvious inhibition in VEGFR1 (Figure S3) and VEGFR2 (Figure S4) expression but also accompanied with an optimal inhibition of VEGF expression both in the mRNA level (Figure 4B) and protein level (Figure 4C, the second column). And, the VEGF mRNA levels (Figure 4B) were measured as 30.3 ± 6.2% in the mice treated with axitinib.

Importantly, axitinib treatment could markedly promote pathological BBB restoration with a prominent increased mRNA level of claudin‐5 (Figure S5A) and occludin (Figure S5B). Compared to the saline‐treated group, the expression of claudin‐5 mRNA was up‐regulated to 48.9 ± 4.6 and occludin mRNA was up‐regulated to 381.6 ± 10.1. In addition, immunofluorescent staining of TJ proteins in axitinib‐treated brain tissues also showed an obvious up‐regulated expression of claudin‐5 (Figure S6A) and occludin (Figure S6C), compared to those of saline‐treated brain tissues. And, the protein expressions of claudin‐5 and occludin were up‐regulated by 4.53‐fold (Figure S6B) and 5.98‐fold (Figure S6D), respectively, compared to the saline group. These results demonstrated that pathological BBB recovery induced by axitinib may serve as an effective strategy for GBM treatment.

### 3.6 Histopathology and immunohischemistry analysis

**FIGURE 5:**
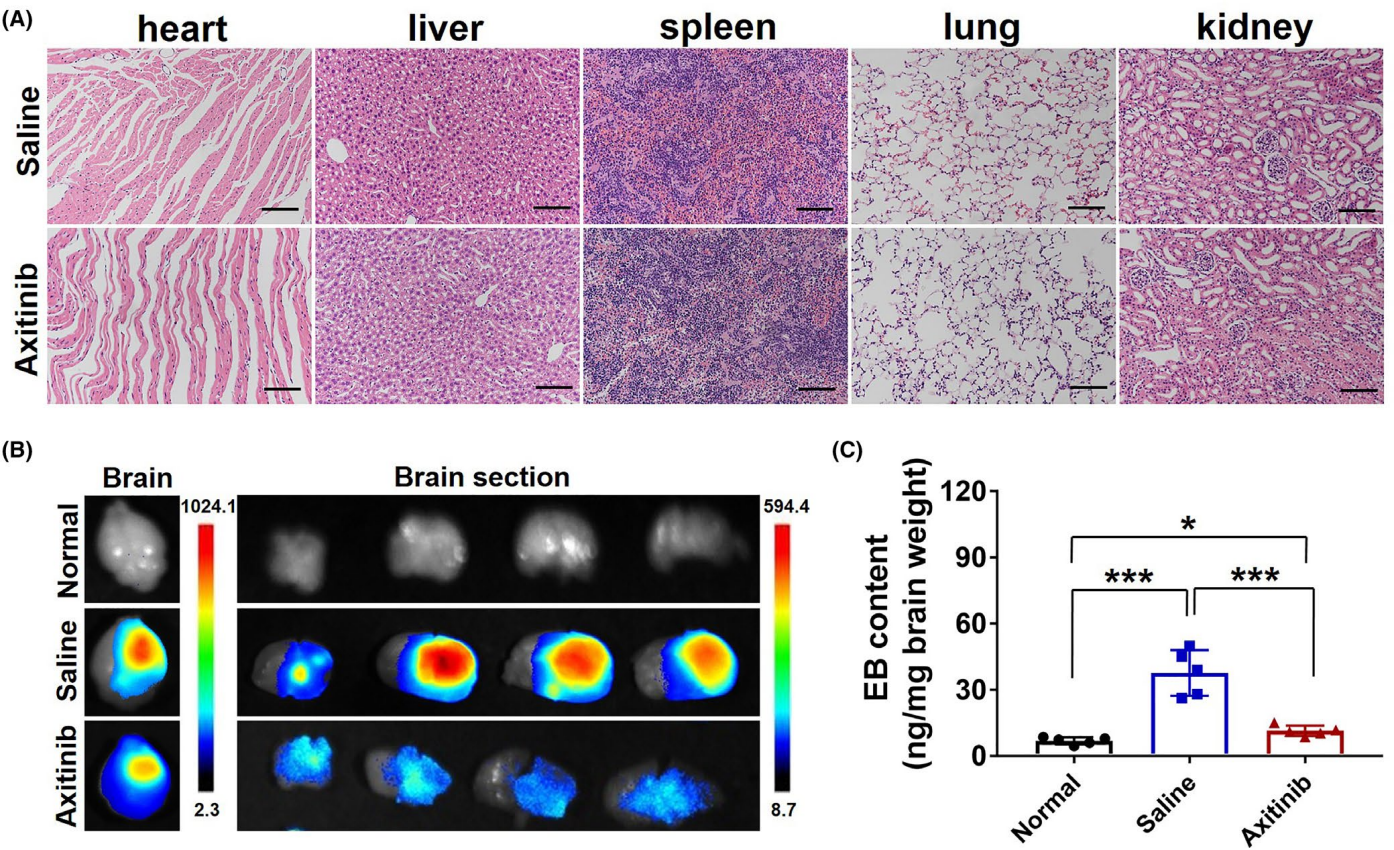
Safety evaluation and EB extravasation. (A) Studies of the long‐term toxicity of different formational drugs in the heart, liver, spleen, lungs, and kidneys using H&E staining. Magnification: 200× (Scale bar = 100 μm). (B) EB extravasation imaging, (C) EB in brain tissues was extracted with dimethyl formamide and quantificationally measured by using an Ultraviolet Spectrophotometer

To further investigate the underlying mechanism of axitinib against GBM, H&E staining and IHC analysis were carried out as shown in Figures 4C and 5A. As shown in Figure 4C, a large amount stroma with compact and regular structure existed in the saline group. In comparison, the structure of stroma in the axitinib group exhibited obviously collapsed, wizened, and vacuous structure. Particularly, necrotic tumor cells exhibiting obvious nuclear chromatin condensation and fragmentation marked by yellow arrows and circled dotted lines appeared after axitinib treatment. Furthermore, CD 31 positive tumor vessels were significantly reduced in axitinib‐treated tumors; in comparison, a greater amount of CD31 positive tumor vessels could be found in tumor tissues treated with saline (Figure 4C, the third column). In addition, MMP‐9 expression (Figure 4C, the fourth column) was also effectively down‐regulated.

Slices of heart, liver, spleen, lungs, and kidneys (Figure 5A) in experimental groups showed no visible abnormalities or lesions compared to those from saline‐treated mice, indicating the lack of appreciable organ damage, further indicating the low toxicity of axitinib.

### 3.7 EB extravasation

Evans Blue extravasation was used to further investigate the antitumor effect of axitinib. Ex vivo imaging (Figure 5B) and quantitative measurement (Figure 5C) results showed that EB extravasation of axitinib‐treated brain was markedly less than other experimental groups, and exhibited no difference to the normal brain. EB content in the brain of the axitinib treatment group decreased 1.5‐fold compared to that of the saline treatment group (Figure 5C). The phenomenon indicated that axitinib could finally produce a decrease in pathological BBB permeability, which would be a good prognosis in GBM therapeutics.

**FIGURE 6:**
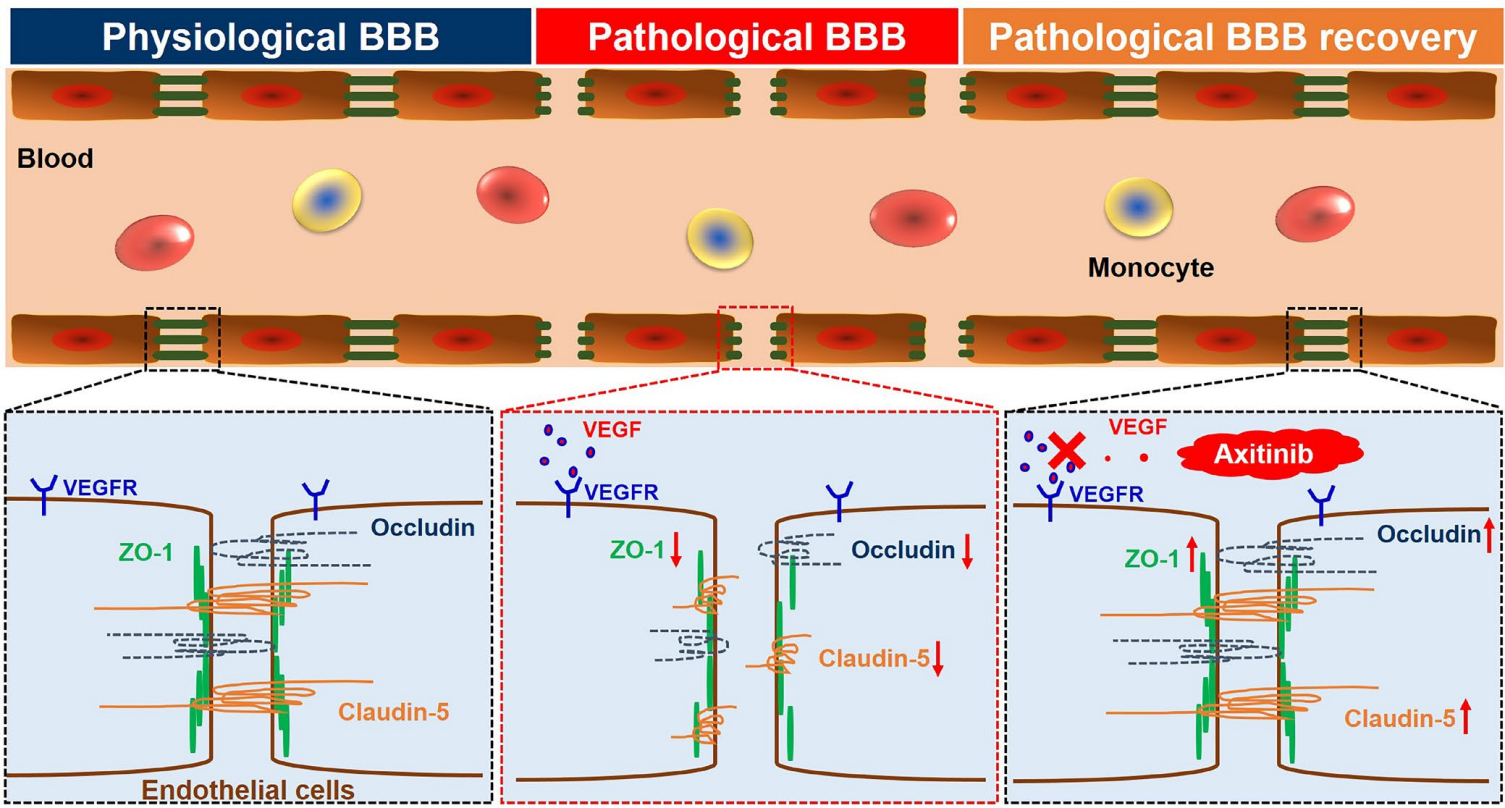
Scheme of BBB functional regulation. The occurrence of GBM induce BBB pathological damage; BBB dysfunction accompanies with TJ protein down‐regulation. Axitinib treatment could restore the pathological BBB; pathological BBB recovery accompanies with an up‐regulated TJ protein expression

The results mentioned above have fully proven that the development of GBM caused severe BBB disruption with TJ proteins down‐regulation. The treatment of axitinib promoted pathological BBB functional recovery with TJ protein up‐regulation, and the restoration of the pathological BBB could finally achieve better therapeutic effects against GBM (Figure 6).

## 4 DISCUSSION

As we know that the BBB is a continuous endothelial membrane playing an important role in the CNS homeostasis regulation,^23^, ^25^ studies have indicated that BBB dysfunction, referring to its loss of structure integrity with increased permeability, has been suggested as a prominent pathological feature of GBM.^26^, ^27^, ^28^ BBB breakdown further facilitates entry into the brain of toxic blood‐derived molecules, cells, and microbial agents, promoting the pathological process and finally leading to poor prognosis.^29^ Since the BBB was shown to be a key component in the brain microenvironment, which is involved in the pathological progress of the diseases, herein, we proposed to regulate pathological BBB function for GBM treatment.

VEGF is confirmed to be abnormally elevated in the pathogenesis of GBM, and the interaction of VEGF and its receptor VEGFR expressed on the cerebral endothelial cells could activate its downstream signaling pathway and then cause BBB dysfunction,^8^, ^30^, ^31^ accompanied with TJ structure breakdown. Therefore, we postulated that inhibiting the combination of VEGF and its receptor would produce a neuroprotection of the pathological BBB. In this study, we assumed the use of axitinib under a non‐cytotoxic dosage to restore the pathological BBB for the abovementioned neurological disorder therapeutics. Interestingly, we have found that axitinib treatment finally produced a significant inhibition in both VEGFR1 (Figure S3) and VEGFR2 expression (Figure S4); meanwhile, the interaction between VEGFR and VEGF was blocked. The suppression of VEGF/VEGFR signal loops finally contributed to pathological BBB restoration accompanied with a prominent increased expression of both claudin‐5 and occludin in the gene level (Figure S5) and protein level (Figure S6). Herein, we speculated that pathological BBB functional recovery induced by axitinib might inhibit nutrient supply between tumor cells and peripheral tissues, finally resulting in GBM growth suppression.

Whether the BBB function is normal or not plays an important role in maintaining the normal activities of the CNS, and also promoting the development of neurological disorders, including GBM.^32^ Since the occurrence and progression of most CNS diseases are accompanied by dysfunction of the BBB, the fact indicates that BBB destruction may be causally related to the occurrence of those brain diseases.^13^, ^33^ Therefore, research on molecular mechanisms involved in BBB functional regulation under both physiological condition and pathological condition has received extensive attention, but there are still many problems that need to be solved. Although it is clear that BBB function is regulated by the brain microenvironment, it remains unknown that BBB disruption is due to the lack of signals maintaining its normal function, or any other abnormal factors caused by different CNS diseases.

Current studies have indicated various factors inducing BBB dysfunction, including special cell secretions in brain tumors.^8^ However, these studies only regarded the damage of the BBB as an index to evaluate the development progress of brain tumors, while ignoring the role of the BBB as a complex and organic part regulating brain functions, including substance transport, transcytosis, and metabolism, as well as TJs. Our study has shown that the occurrence of GBM induces BBB pathological damage, accompanied with TJ disruption; moreover, the functional restoration of the pathological BBB via TJ regulation could improve the brain microenvironment and alleviate the progression of the disease (Figure 6). Therefore, it is, indeed, time to change our minds. The BBB is not just a non‐fenestration barrier protecting the brain from the external environment, but also an organism with complex and sophisticated properties and functions, even an organoid as Richard Daneman put forward.^34^ It is a serious barrier for intracranial drug delivery. In order to investigate the function of the BBB and its regulatory mechanism under physiological and pathological conditions, we need to make a more in‐depth comparative study and analysis by using multi omics technology combined with different disease models. In addition, we also need to consider how the BBB interacts with other tissues and organs in the body at a higher organoid level to regulate the homeostasis of the whole body. These are not only the lack of this research, but also our future research direction.

In conclusion, we demonstrated for the first time the effect of axitinib recovering the pathological BBB for GBM treatment. Axitinib under non‐cytotoxic dosage regulated pathological BBB function recovery in a time‐dependent mode, and multiple interventions of axitinib could realize a visible restoration of the pathological BBB in vitro. The use of axitinib finally produced considerable treatment effect on GBM. Our study is the first to demonstrate that recovering the pathological BBB would produce visible therapeutic effect to GBM relating to BBB permeability. The study supports further investigation of axitinib as a BBB permeability regulator for other neurological disorder treatments. Furthermore, the view of regulating pathological BBB functional recovery may offer a novel sight for other CNS disease therapeutics.

## CONFLICT OF INTEREST

No potential conflicts of interest were disclosed.

## Supporting information

Supplementary Material

## Data Availability

The original contribution presented in the study is included in the article/Supplementary Materials. Future inquiries can be directed to the corresponding author.
