# Supplementary material for: Effect of axitinib regulating the pathological blood–brain barrier functional recovery for glioblastoma therapeutics
Source: CNS Neurosci Ther. 2021 Dec 29;28(3):411–21. doi: 10.1111/cns.13788 (PMC8841308; doi:10.1111/cns.13788)

**Supporting Information**

**Effect of axitinib regulating pathological blood brain barrier functional recovery for glioblastoma therapeutics**

Fengtian Zhang ^1, 2,^ *｜Lijuan Wen ^3, 4,^ *｜Kai Wang ^3,^ *｜Zhihua Huang ^5, 6^｜Xiangyu Jin ^3^｜Ruiwen Xiong ^4^｜Shiying He ^4^｜Fuqiang Hu ^3^

^1^ Department of Orthopedics, Second Affiliated Hospital of Soochow University, 181 Sanxiang Road, Gusu District, Suzhou 215004, China.

^2^ Department of Orthopedics, First Affiliated Hospital of Gannan Medical University, Jinling East Avenue, Zhanggong District, Ganzhou 341000, China.

^3^ College of Pharmaceutical Sciences, Zhejiang University, 866 Yuhangtang Road, Hangzhou 310058, China.

^4^ National Engineering Research Center for Modernization of Tranditional Chinese Medicine-Hakka Medical Resources Branch, College of Pharmacy, Gannan Medical University, University Park in Rongjiang New District, Ganzhou 341000, China.

^5^ Institute for Medical Sciences of Pain, Department of Physiology, School of Basic Medical Sciences, Gannan Medical University, University Park in Rongjiang New District, Ganzhou 341000, China.

^6^ Key Laboratory of Prevention and treatment of cardiovascular and cerebrovascular diseases of Ministry of Education, Gannan Medical University, University Park in Rongjiang New District, Ganzhou 341000, China.

* These authors contributed equally to this work.

**Correspondence**

Fuqiang Hu, College of Pharmaceutical Sciences, Zhejiang University, 866 Yuhangtang Road, Hangzhou 310058, China. Email: hufq@zju.edu.cn.

**METHODS**

**Cell models**

For the establishment of the *in vitro* physiological BBB model, the bEnd.3 cells were seeded on polycarbonate filter membranes with a pore size of 0.4 μm and a surface area of 1.12 cm^2^ (Costar Transwell, Millipore Corp., Bedford, MA, U.S.A.) at a density of 10 × 10^4^ cells per filter. During the culture for 15 days, the medium in both upper and lower compartments were changed every other days, and the integrity of cell monolayer was verified by measuring the transepithelial electrical resistances (TEER>150 Ω•cm^2^) values using a Millicell-ERS volt-ohmmeter (Millipore Co., U.S.A.). A bEnd.3 cells and U87 MG cells co-cultured model was established to imitate pathological BBB. U87 MG cells were seeded into 12-well plates at a density of 15 × 10^4^ cells/cell at 37 ℃ for 12 h to attach, then the inserts with monolayer bEnd.3 cells with feasible TEER were transferred to the plates with confluent U87 MG cells. The two kinds of cells in the transwell-chambers were co-culture for another 24 h.

After incubation, bEnd.3 cells and U87 MG cells were washed with HBSS for three times and pre-treated with HBSS for 30 min at 37 ℃ before the experiments. For TEER measurement, the mode of Millicell-ERS volt-ohmmeter was switch to ohms and power on, then the electrode was inserted into the Millicell Insert with long electrode on the outside and the short electrode on the inside, and the electrode must be perpendicular to the petri dish. TEER was calculated using the following formula: TEER = R × surface area (1.12 cm^2^).

**Animal models**

For the orthotopic GBM model, male BALB/C nude mice (18-20 g) were intracranial injected with U87-luci cells (5 × 10^5^ cells in 5 μL of serum-free culture medium) at the right striatum of each male nude mice (2 mm lateral to the bregma, 0.8 mm anterior to the bregma and 3 mm deep from the dura) at a rate of 1.0 μL/min using a stereotaxic apparatus equipped with a mouse adapter (RWD Life Science Co., Ltd, Shenzhen, China). All animal experiments were bred and maintained in a specific pathogen free barrier facility and all procedures were approved and performed in accordance with national guidelines of the ethical committee of Zhejiang University. All surgeries were performed under general anesthesia, and all efforts were made to minimize adverse effects.

**Cytotoxicity assay**

bEnd.3 cells/U87 MG were seeded into 96-well plates at a density of 8 × 10^3^ cells per well in 200 μL of complete medium. After 12 h incubation, the cells were exposed to serial concentrations of axitinib in the range of 0-10 μg/mL at 37 ℃ for 48 h. At the end of incubation, 20 μL of MTT solution (5 mg/mL in deionized (DI) water) was added into each well and incubated at 37 ℃ for another 4 h, then the media was replaced with 200 μL DMSO to dissolve the purple formazan crystals. After shaking for 15 min, the absorbance of each well was measured by a micro plate reader (Model 680; BioRad, Hercules, CA) at 570nm.

**Immunofluorescent staining of tight junctions proteins**

bEnd.3 cells covered onto the polycarbonate membrane of transwell inserts were washed with PBS and fixed with 4% formaldehyde, then blocked with 10% BSA for 30 min at 37 ℃. The cells were then incubated with anti-Claudin-5 primary antibodies (1:40 dilution; Abcam) and anti-Occludin primary antibodies (1:50 dilution, Abclonal) overnight at 4 ℃ followed by incubation with secondary antibodies of goat anti-rabbit DyLight 649 and goat anti-rabbit DyLight 488 (1:200 dilution; Multi Sciences), respectively. The immunofluorescence images were obtained by CLSM on an x-y mode.


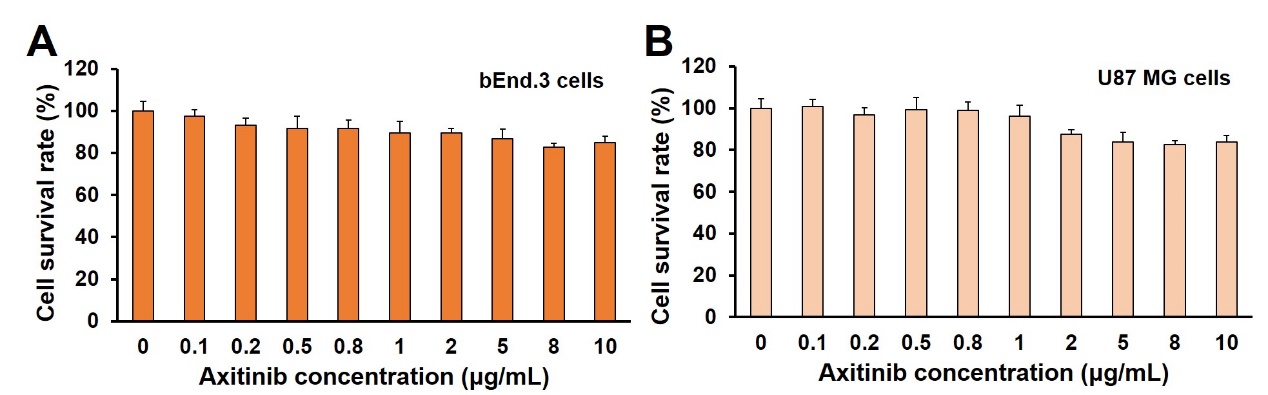


**FIGURE S1** Cytotoxicity of axitinib. *In vitro* cell viability of (A) bEnd.3 cells and (B) U87 MG cells after treated with a concentration of 0-10 μg/mL axitinib-equiv./mL for 48 h. Error bars represent SD of the mean for n = 5.


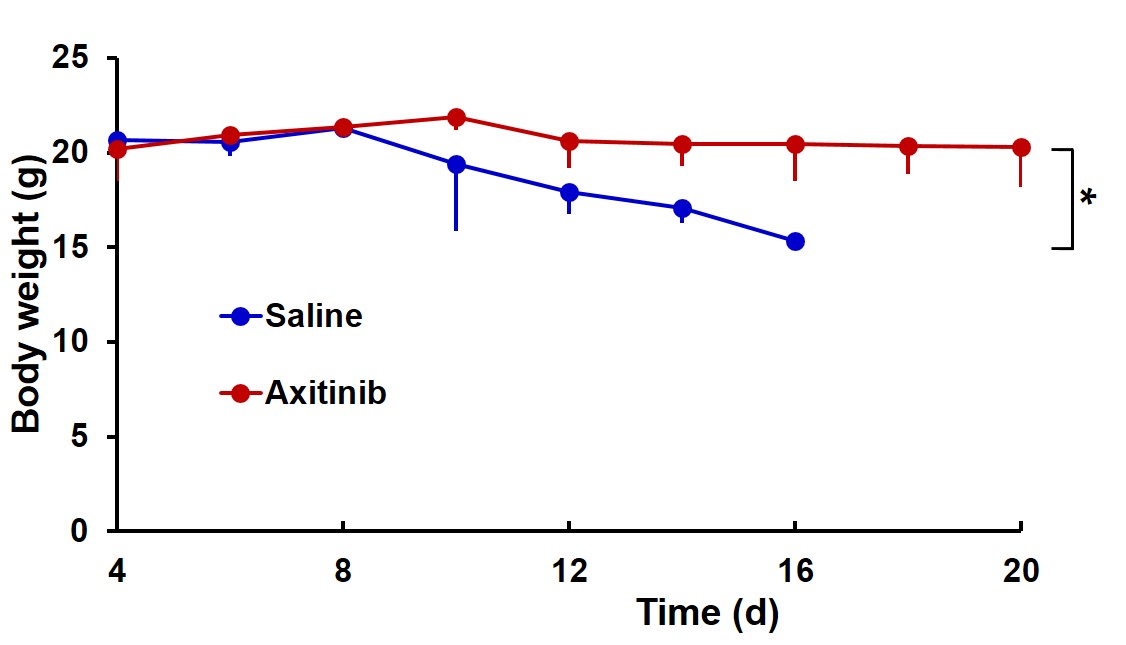


**FIGURE S2** Body weight curves of GBM-bearing animals. Body weight were measured every two days after the first injection (n = 6).


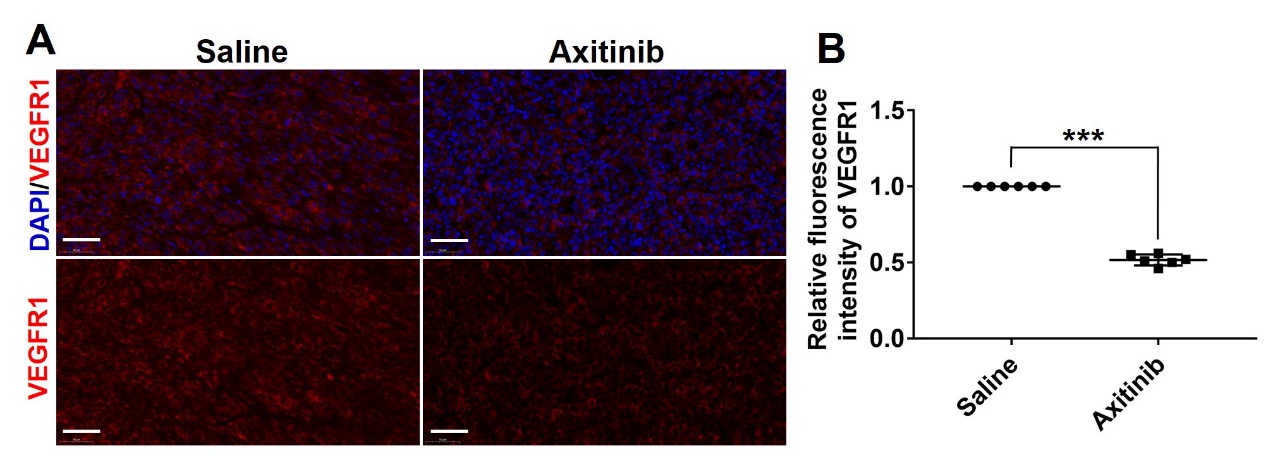


**FIGURE S3** VEGFR1 expression. (A) VEGFR1 expression in GBM-bearing brain tissues after axitinib treatment and (B) the fluorescence semi-quantitative analysis evaluated by Image J.


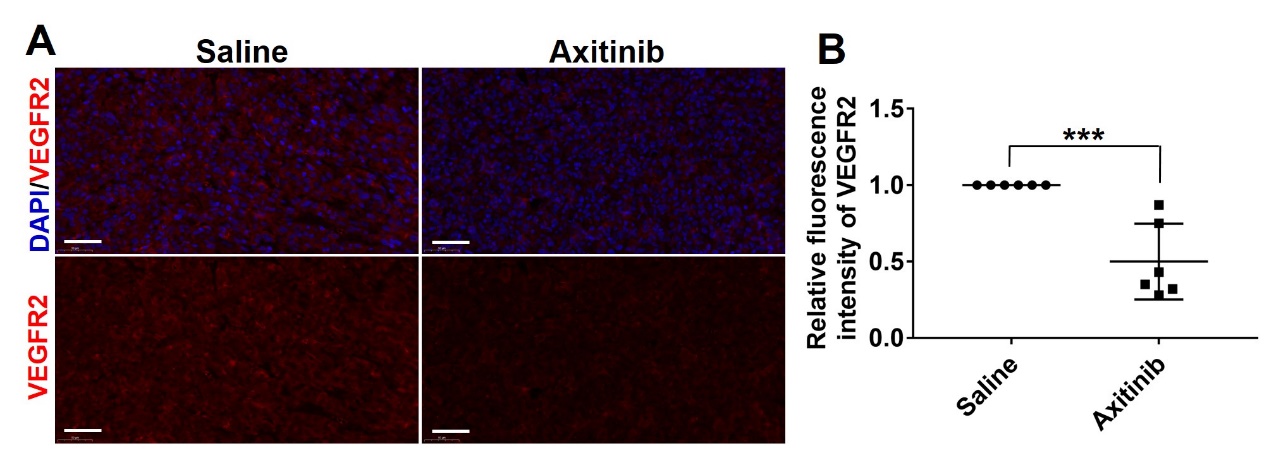


**FIGURE S4** VEGFR2 expression. (A) VEGFR2 expression in GBM-bearing brain tissues after axitinib treatment and (B) the fluorescence semi-quantitative analysis evaluated by Image J.


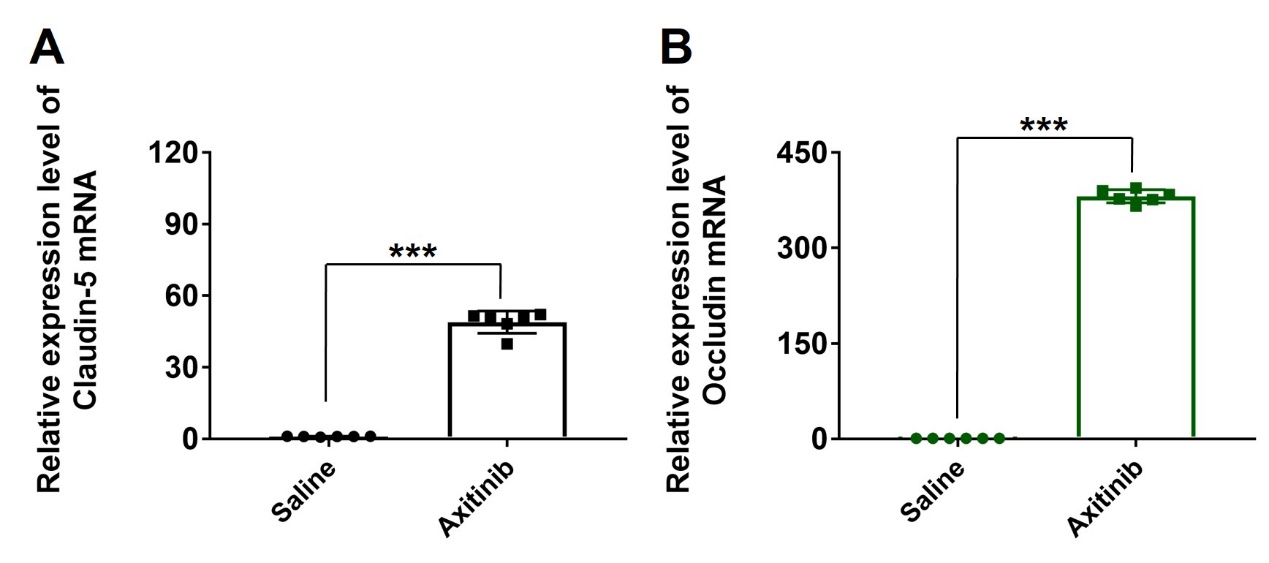


**FIGURE S5** Gene expression for TJs proteins. (A) Claudin-5 mRNA and (B) Occludin mRNA expression of tumor tissues after axitinib treatment. Data were expressed as the fold change in Claudin-5 and Occludin expression normalized with the housekeeping gene, respectively, and GAPDH was amplified as an internal control.


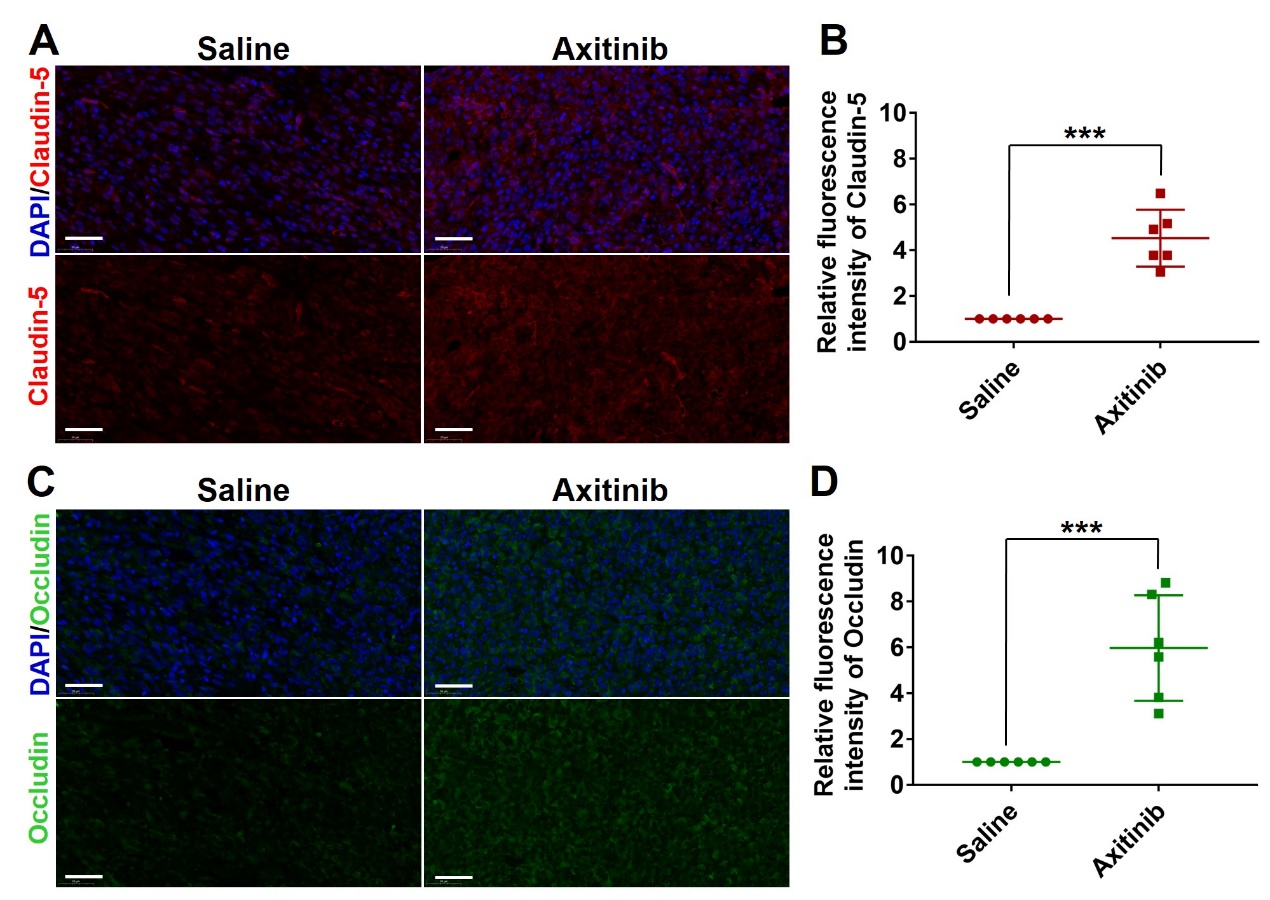


**FIGURE S6** TJs proteins expression. (A) Claudin-5 (red) expression in GBM-bearing brain tissues after axitinib treatment and (B) the fluorescence semi-quantitative analysis evaluated by Image J. (C) Occludin (green) expression in GBM-bearing brain tissues after axitinib treatment and (D) the fluorescence semi-quantitative analysis evaluated by Image J.

**TABLE S1** Quantitative analysis of orthotopic GBM growth. Relative ROI change fold in tumor bioluminescence throughout the course of orthotopic GBM treatment (n = 5).


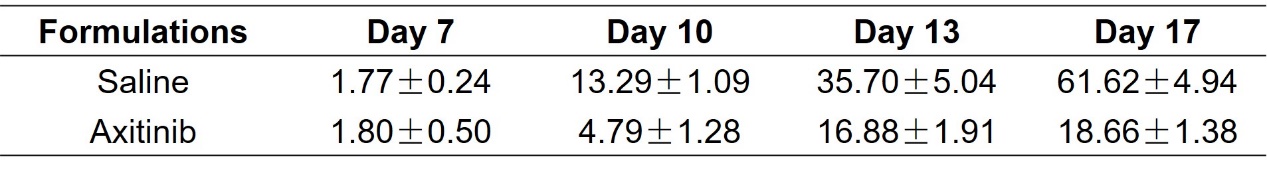

Supplement: Supplementary file 1 — Supplementary Material [file CNS-28-411-s001.docx]
